# Circulating tumor DNA detection in head and neck cancer: evaluation of two different detection approaches

**DOI:** 10.18632/oncotarget.20004

**Published:** 2017-08-07

**Authors:** Sandra Perdomo, Patrice H. Avogbe, Matthieu Foll, Behnoush Abedi-Ardekani, Violeta Lescher Facciolla, Devasena Anantharaman, Priscilia Chopard, Florence Le Calvez-Kelm, Marta Vilensky, Jerry Polesel, Ivana Holcatova, Lorenzo Simonato, Cristina Canova, Pagona Lagiou, James D. McKay, Paul Brennan

**Affiliations:** ^1^ International Agency for Research on Cancer (IARC), Lyon 69372, France; ^2^ Institute of Nutrition, Genetics and Metabolism Research, Faculty of Medicine, Universidad El Bosque, Bogotá 110121, Colombia; ^3^ Departamento de Saúde Coletiva, Faculdade de Ciências Médicas da Santa Casa de São Paulo, São Paulo CEP01221-020, Brazil; ^4^ Instituto Angel Roffo, Buenos Aires C1417DTB, Argentina; ^5^ CRO Aviano National Cancer Institute, Aviano 33081, Italy; ^6^ Charles University, 1st Faculty of Medicine, Prague 116 36, Czech Republic; ^7^ Laboratory of Public Health and Population Studies, University of Padova, Padova 35122, Italy; ^8^ Department of Hygiene, Epidemiology and Medical Statistics, School of Medicine, National and Kapodistrian University of Athens, Athens 11527, Greece; ^9^ Current present address: Rajiv Gandhi Centre for Biotechnology, Trivandrum 695010, India

**Keywords:** ctDNA, head and neck, cancer, mutation, detection

## Abstract

The use of non-invasive biomarkers such as circulating tumor DNA (ctDNA) in head and neck tumors may be of relevance in early diagnosis and eventually improved outcome. We evaluated two different approaches from two case series in Europe and South America including (i) targeted screening of ctDNA mutations, and (ii) detection of *TP53* mutations in plasma and oral rinses without previous knowledge of mutational status in tumor samples. Targeted sequencing in 5 genes identified ctDNA mutations in plasma among 42% of HNSCC cases, 67% of who were early stage cases. No association was found between ctDNA mutation detection and overall survival. Sequencing of the entire coding region of the *TP53* gene resulted in identification of *TP53* mutations in 76% of tumor cases. However, concordance of mutation detection was low between tumor, oral rinses (11%) and plasma (2,7%) samples. Identification of 5 pathogenic *TP53* mutations in oral rinses from 3 non-cancer controls gives additional evidence of mutation occurrence in individuals without a diagnosed cancer and presents an additional challenge for the development of ctDNA diagnostic assays.

## INTRODUCTION

Cell-free DNA (cfDNA) are small nucleic acid fragments released in body fluids and their levels are a reflection of the dynamics between cellular mechanisms of DNA release and DNA clearance, apoptosis and necrosis, cfDNA stability, blood nuclease activity and uptake and degradation by phagocytes [1, 2]. Among individuals with cancer a fraction of cfDNA is potentially of tumor origin, i.e. cfDNA fragments harboring tumor somatic nucleic acid changes that are also called circulating tumor DNA (ctDNA). Some studies have correlated the abundance of ctDNA with tumor size and stage, with as much as 40% of ctDNA levels in metastatic cancers but as low as 0.1-1% in premalignant or early stage disease [3–8]. Clinicopathological characteristics for specific tumor types, such as anatomic location, tumor grade, tumor mucinous features and treatment response [4, 9, 10] might also play an important role in the process of cfDNA release and stability in blood circulation.

Head and neck squamous cell carcinomas (HNSCC) are the seventh most common cause of death in the world accounting for approximately 375,000 cancer deaths annually. In 2012, almost 700,000 new head and neck cancer cases were estimated to occur worldwide [11]. Despite current therapeutic interventions, the prognosis for HNSCC is relatively poor, with a 5-year survival ranging from approximately 25% to 60%, according to cancer subsite [12]. Diagnostic delay is a recognized challenge for patients with HNSCC, and has also been related to higher risk (30%) of advanced stage tumor diagnosis eventually impacting negatively on prognosis and survival [13]. These delays could be shortened in many patients through the examination of clinically suspicious lesions using non-invasive biomarkers such as ctDNA. In HNSCC patients, besides circulating DNA fragments carrying tumor alterations in plasma, the saliva contains a high fraction of tumor DNA due to its close contact to oral cavity and pharyngeal tumor sites constituting an additional biological source for ctDNA detection and analysis [14–16].

Our primary goals were to provide a comprehensive evaluation of the presence of ctDNA in plasma and oral rinses from a series of HNSCC cases at early and late stages and to determine the best approach to use ctDNA analysis in HNSCC early detection. We evaluated two strategies of ctDNA mutation detection. A targeted approach tested for the presence of mutations in plasma samples, previously detected in tumor samples. The second approach involved ctDNA screening detection in plasma and oral rinses without prior knowledge of mutation status in tumors.

## RESULTS

### ctDNA targeted detection in plasma samples from the ARCAGE study

**Figure 1:**
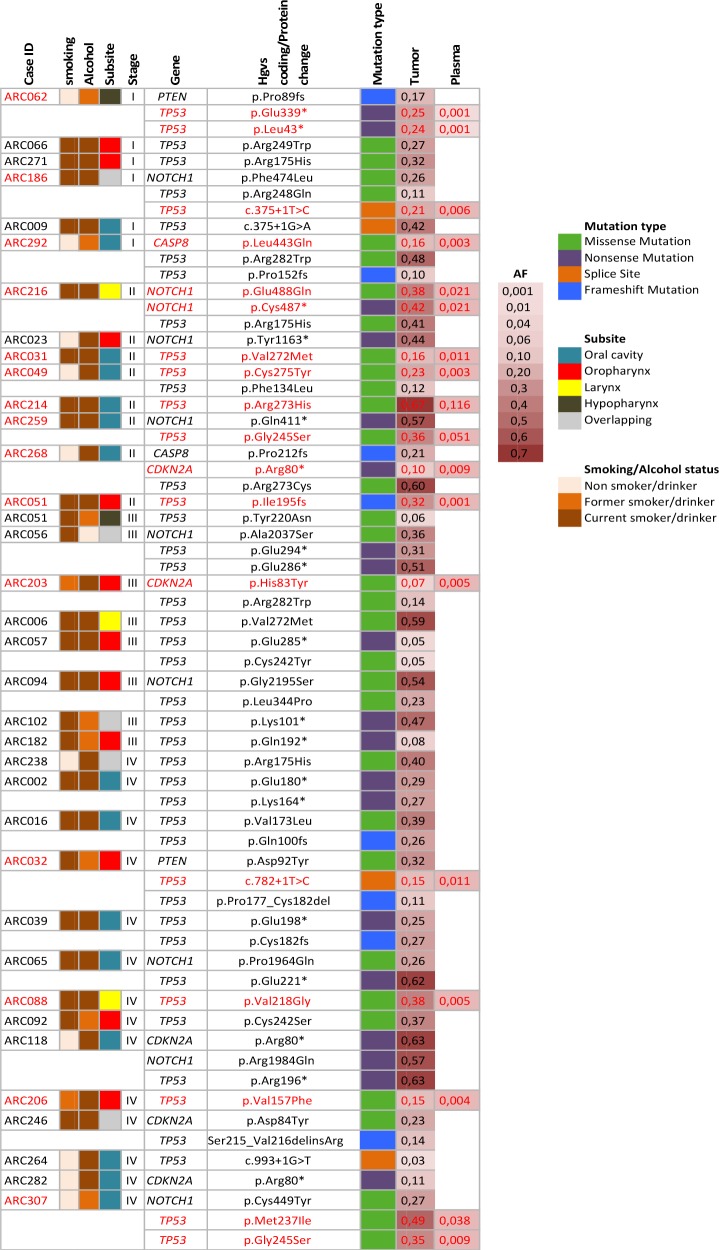
Targeted mutation detection in plasma of 36 HNSCC cases from the ARCAGE study Description of clinical and epidemiological characteristics of cases and ctDNA mutation detection in plasma. Cases are organized by stage. In red, mutations identified in matched tumor and plasma samples. AF: mutation allelic frequency.

36 HNSCC cases from the ARCAGE study were selected based on carrying one or several mutations in their tumor samples in the 5 screened genes (*TP53, NOTCH1, CDKN2A, CASP8, PTEN*) (Figure 1). All cases were HPV negative. Forty-two percent of cases (15/36) had detectable ctDNA mutations in their plasma samples, sixty seven percent of those were early stage (I, II) cases. A total of 18 mutations, previously detected in the matched tumor samples, were detected in plasma with allelic fractions (AF) ranging from 0,001-0,12 (Figure 1). Among those mutations, we were able to detect a *TP53* 16-nucleotide frameshift deletion in a stage II oropharyngeal case (Supplementary Figure 1). Cases with identified plasma mutations showed no differences in overall survival compare to those without ctDNA mutations (Log rank p=0,47).

### TP53 mutation detection in tumor, plasma and oral rinses of HNSCC cases from the LA study

**Figure 2:**
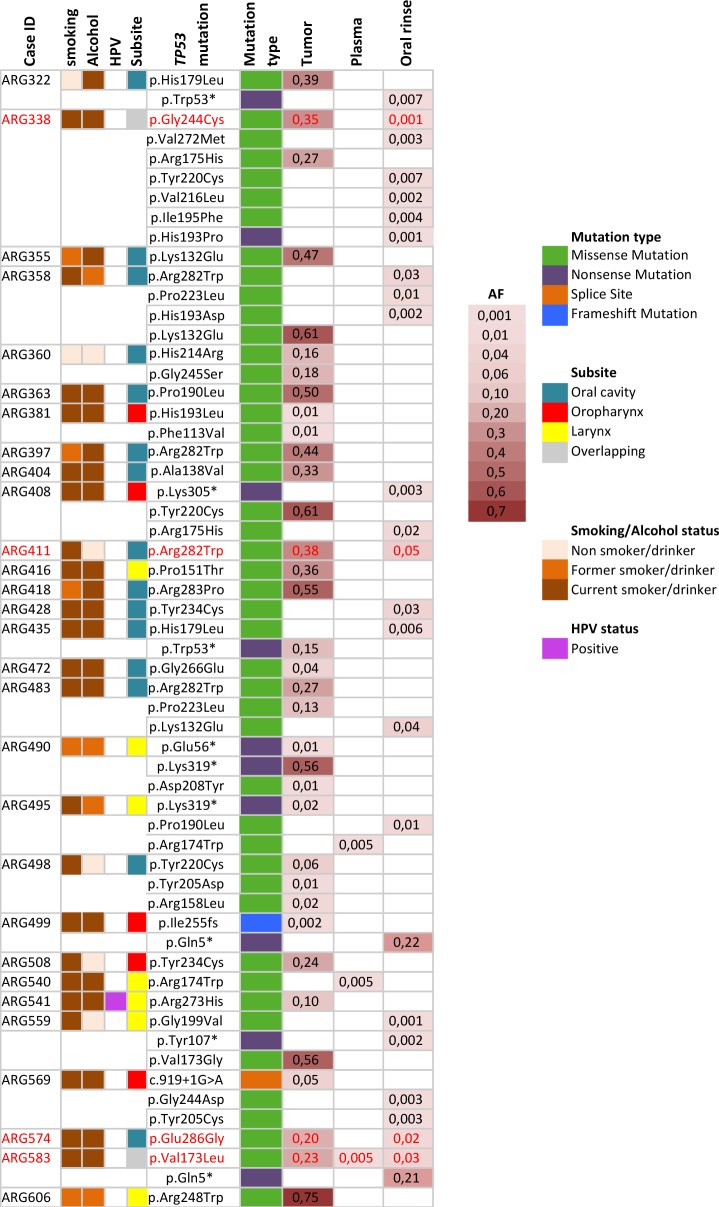
Description of *TP53* mutations identified in Tumor, plasma and oral rinses from a series of 37 cases from the LA study Only cases with *TP53* mutations are shown. In red, mutations identified in matched tumor and oral rinses or plasma samples. AF: average allelic frequency from both libraries.

**Figure 3:**
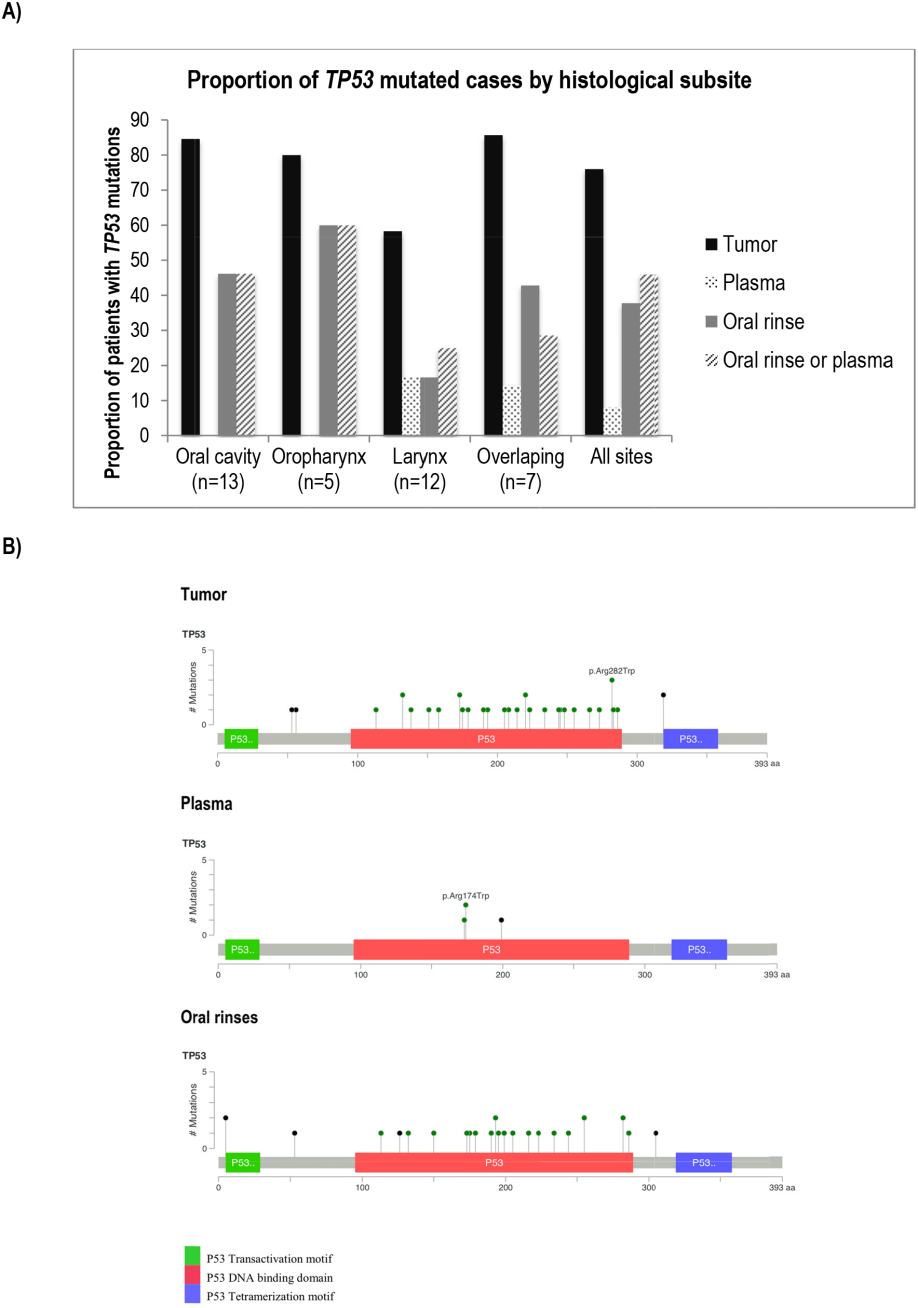
Comparison of *TP53* mutations found in tumor, plasma and oral rinses from a series of 37 cases from the LA study **(A)** Proportion of cases harboring *TP53* mutations in tumor, plasma and oral rinses by subsite. **(B)** Diagrams of mutation distribution along the *TP53* coding regions and protein domains. Mutation colors represent: green: Missense Mutations; black: Truncating Mutations (Nonsense, Nonstop, Frameshift deletion, Frameshift insertion, Splice site).

We performed independent *TP53* mutation detection analyses for the 37-late stage HNSCC cases in each sample type: tumor, plasma and oral rinses. A total of 36 *TP53* mutations were identified in tumors, 3 in plasma and 26 in oral rinses. Seventy-six percent (28/37) of cases harbored at least one *TP53* mutation in the tumor sample. One case (ARG5040) had a mutation (*TP53* p. Arg174Trp) found exclusively in plasma (Figure 2). We found no association between age, subsite, smoking or alcohol status and the presence of *TP53* mutations in tumors (Supplementary Table 1 and Figure 3B). The proportion of cases with ctDNA *TP53* variants in oral rinses was higher in patients with tumors in the oral cavity 46,2% (n=6) and in the oropharynx 60% (n=3) than in the Larynx 16,7% (n=12) (Figure 3A). More than 80% of overall mutations corresponded to missense variants, all localized in the DNA binding domain (aminoacids 102-292) and 86% were non-functional variants according to the functional classification based on the overall transcription activity [17] (Figure 3B and Supplementary Table 2). The mutational distribution along the coding regions and protein domains was comparable for all three sample types (Figure 3A). Four cases showed concordance in mutation detection between tumor and oral rinses and only in one additional case, we identified the same mutation in tumor, plasma and oral rinses (Figure 2). Fourteen cases (52%) showed detectable *TP53* mutations in tumor but none in plasma or oral rinses.

### TP53 mutation detection in non-cancer controls

One additional finding was the observation that *TP53* mutations could be detected in the plasma and oral rinses of a few non-cancer individuals. We identified 5 *TP53* mutations in oral rinses from 3 individuals without cancer diagnosis with AFs ranging from 0,001-0,004. All 5 mutations were classified as pathogenic and located in the DNA binding domain. Two of those individuals were both never smokers and non-drinkers (Table 1).

**Table 1:** Description of *TP53* mutations found in oral rinses of 3 Argentinian controls with the mutations’ allelic fractions detected in the two libraries

Control ID	Disease classification (ICD-10-CM)	Coverage Library 1	Coverage Library 2	Allelic Fraction Library 1	Allelic Fraction Library 2	Chr	Start	End	Ref	Alt	Mutation Type	Cosmic-76	Hgvs DNA Change	Hgvs Protein Change	Transactivation Class
ARG553	Hydrocele and spermatocele	35483	36558	0,001	0,002	17	7577530	7577530	T	A	Missense Mutation	COSM43967	c.614A>G	p.Ile251Phe	non-functional
ARG410*	unspecified disorders of male genital organs	28491	38571	0,004	0,003	17	7577548	7577548	C	T	Missense Mutation	COSM1640833	c.661G>A	p.Gly245Ser	supertrans
ARG594*	Osteoarthritis	29062	27304	0,001	0,001	17	7577609	7577609	C	G	Splice Site	COSM562645	c.673-1G>C	NA	NA
		37034	21337	0,002	0,002	17	7578188	7578188	C	T	Missense Mutation	COSM44853	c.733G>A	p.Glu221Lys	non-functional
		39098	22393	0,004	0,004	17	7578235	7578235	T	C	Missense Mutation	COSM99631	c.751A>T	p.Tyr205Cys	non-functional

NA: Not applicable. Chr: Chromosome.

* both non-smokers non-drinkers.

As was reported before by us, 5 *TP53* mutations were found in the plasma of 4 of the non-cancer individuals analyzed from Argentina, one being confirmed to be a germline variant [18].

## DISCUSSION

Targeted ctDNA screening is an increasingly used non-invasive method for mutation detection in human cancer, both for diagnosis and disease monitoring. Recently, it has been shown that screening for specific mutations in plasma and saliva of HNSCC patients is a sensitive and specific approach [4, 14]. In our series of 36 cases from the ARCAGE study, we detected specific tumor-concordant mutations in 5 genes in the plasma of 42% of cases and the majority of those (67%) were early-stage cases. However, our overall mutation detection rate in plasma was low as we were able to detect only 28% of the total number of mutations tested. Limitations regarding sample preservation might account in part for the low mutation detection rate in plasma. In particular, cfDNA degradation due to prolonged storage is one of the pre-analytical variables that have an impact on cfDNA recovery and mutation detection, and recent studies recommend the use of plasma samples or cfDNA extracts up to nine months of storage at −20°C or −80°C [19]. Additionally, preanalytical recommendations for ctDNA analysis indicate that blood samples can be kept up to 4 hrs at room temperature (RT) before plasma preparation without major impact on cfDNA concentration [19]. The protocols for blood recovery and processing followed by both studies (ARCAGE and LA) allowed a time window for blood processing up to 12 hrs at RT, which could have resulted in a reduction of cfDNA concentration before plasma storage. This is the first study evaluating ctDNA using retrospective HNSCC samples from a case series where plasma samples exceeded 10 years of storage at −80°C. Fifty percent of mutation detection in long stored plasma has been only previously reported for *KRAS* mutations identified by Sanger sequencing in plasma stored up to 6 years from metastatic colorectal patients [20].

Analysis of ctDNA detection and survival in the ARCAGE cases found no association between ctDNA mutation status and HNSCC survival overall. No additional studies have previously evaluated the impact in survival of ctDNA detection in head and neck cancer cases limiting the comparability of our results. Association between ctDNA detection at diagnosis and survival is consistent across several metastatic and advanced tumor types [5, 21–23]. However, limited data is available on early stages and while ctDNA detection has been found to be associated with poor overall survival in some early stage pancreatic and colorectal cases [7, 24] no difference was observed in early breast cancer cases [8]. Future prospective studies are then necessary to evaluate the prognostic clinical significance of ctDNA detection in both early and late stage HNSCC cases.

While targeted sequencing might be sufficient for post-diagnosis uses of ctDNA, in the case of early detection it might be necessary to interrogate the whole coding region of the most frequently altered genes in HNSCC rather than focusing on selected targets. The mutational profile of HNSCC is characterized by recurrent alterations in tumor suppressor genes (of the 15 most common HNSCC mutated genes only *HRAS* and *PIK3CA* are oncogenes) where singular hot spots are infrequent and the mutational spectra covers various exon regions and/or specific functional protein coding domains [25]. Consistently with previous reports, 76% of the tumor cases from the LA study analyzed harbored a *TP53* mutation [25, 26], which makes *TP53* one of the suitable biomarkers for non-invasive early detection of HNSCCs. Yet, the concordance in *TP53* mutation detection between tumor and oral rinses was 11% for tumors located in the oral cavity and overlapping sites. The salivary genome consists of various DNAs representing the genome of an individual, oral microbiota and infecting DNA viruses. Such diversity can undermine tumor DNA detection while increasing the mutational background. However, the quality and yield of DNA that can be obtained from saliva as well as its stability for long-term storage might compensate this limitation and might make it a robust analyte choice for diagnostics [15, 27]. In this regard, Wang and collaborators identified mutations in 6 commonly mutated genes for HNSCC in both saliva and plasma of 93 patients and reported 100% sensitivity of ctDNA detection in saliva specimens in tumors of the oral cavity [14]. Furthermore, the authors found that both the sensitivity and the fraction of mutant alleles decreased in HNSCCs distal to the oral cavity which agrees with the higher percentage of mutations we have found in both oral cavity and oropharynx compared to laryngeal cases. *TP53* mutations have been previously identified in 23,5% of oral rinses from cases with homogeneous oral leukoplakia (OL) and in 40% of OL cases with an earlier diagnosis of one or several oral squamous cell carcinomas [28]. One additional study, based on microsatellite marker detection in tumor and exfoliated oral mucosal cells, detected tumor DNA in 44% of oral rinse samples [16]. Variations in tumor DNA detection frequencies between these studies might be due to the use of different mutation detection techniques (massive parallel sequencing or Sanger sequencing).

Interestingly, we found 22 additional mutations in oral rinses from 11 cases not found in their matched tumor samples but identified in similar coding regions (aminoacids102-292) and with the same pattern of mutational distribution along the *TP53* protein domains as those found in tumor samples. The additional burden of mutation in oral rinses might reflect tumor heterogeneity (especially in the case of advanced tumors) where sub clonal populations of cells are not necessarily captured by tumor biopsies and/or could reflect genetic alterations in the squamous epithelial cells lining the oral cavity as a result of field cancerization, mostly associated to smoking and alcohol consumption. Evidence of mutational heterogeneity in the oral epithelium of HNSCC cancer patients has been recently reported. Wood and collaborators showed the presence of discordant *TP53* mutations in dysplastic tissue and adjacent cancer tissue from the same patient. They also reported the presence of different mutations in other genes at very low allelic frequencies (<0,05-0,2) reflecting sub clonal cell populations present in either adjacent or distant dysplastic tissue but absent in the pair tumor tissue [29].

Our percentage of mutation detection in plasma in cases from Argentina (LA study) was much lower (8,1%) compared to oral rinses (40,5%, all sites) and restricted to cases in larynx and overlapping sites. These results indicate that the impact on ctDNA detection after long storage of plasma (discussed above) had a higher effect on cases from Argentina (8,1% ctDNA plasma detection) since these samples were collected 4 years before samples from the ARCAGE study (28% ctDNA plasma detection).

Finally, identification of *TP53* mutations of non-cancer individuals introduces new questions regarding the biological role of such pathogenic mutations in healthy individuals and challenges the specificity of ctDNA screening in possible diagnostic scenarios. Although detection of *TP53* mutations in plasma, human skin and peritoneal fluid of healthy individuals has been reported previously [18, 30–32], we detected for the first time, 5 pathogenic *TP53* mutations in oral rinses from 3 non-cancer individuals. However, one limitation of our results is that non-independent germline sample (buffy coat) was not available to confirm the somatic origin of these mutations. In fact, the presence of circulating-mutated fragments resulting from clonal hematopoiesis in healthy individuals has been well documented [33–38]. Most recently, in 53105 individuals without a known cancer diagnosis from the Exome Aggregation Consortium, 2,7% germline DNA repair mutations were identified [39]. Without discarding the plausibility of these mutations being germline alterations, the low allelic fraction in the mutations detected in oral rinses (0,001-0,004) suggests that they may well correspond to somatic changes in the oral mucosa. A recent study has reported a higher somatic mutation burden in 9 head and neck non-malignant tissues compared to 61 benign tissues [40]. Since we used hospital-based controls as a proxy for healthy individuals, mutations identified might reflect biological processes underlining inflammatory responses (for 2 never smoker, non-drinker controls) and/or the smoking and alcohol accumulated effect in the oral mucosa.

Together, our findings confirm the feasibility of ctDNA targeted mutation detection in plasma of HNSCC patients including early stages. Blind detection of *TP53* mutations in oral rinses or saliva had a low concordance compared to mutations identified in the matched tumors. Identification of 5 *TP53* mutations in oral rinses from healthy controls confirms the presence of a small percentage of pathogenic mutations in healthy individuals, a fact that should be taken into consideration when developing diagnostic ctDNA assays for early HNSCC detection. Further prospective studies are necessary in order to define both the diagnostic value and prognostic clinical significance of ctDNA detection in HNSCC.

## MATERIALS AND METHODS

### Study population and mutation detection design

Cases and controls were selected from two multicentre studies: one conducted in South America (LA study) between 1998 and 2002 and the second between 2002 and 2005 in Europe (ARCAGE study), from which biological samples were available in the IARC biorepository, along with complete epidemiological data. Extensive details on data and sample collection from these studies are included elsewhere [41–43]. Briefly, all subjects underwent personal interviews to collect information on lifestyle exposures and hospital records were reviewed to obtain clinical and pathological information [41, 42]. Centralized HPV testing was completed for both studies determined on serology testing as described before [44]. HPV positivity was defined based on HPV16 E6 status, which has been shown to be a highly sensitive and specific marker of HPV16-related oropharyngeal tumours [43, 45, 46]. Informed consent was obtained from all participants in the two studies, and the analysis was approved by the Ethical Review Committee of the International Agency for Research on Cancer.

In order to test the targeted ctDNA detection approach, we selected 36 HNSCC fresh tumor samples, including 14 early (stage I and II) cases, from the ARCAGE study with available matched plasma samples. Oral rinses were not collected as part of the ARCAGE study [41]. DNA from 36 HNSCC tumors had been previously sequenced and found to carry 65 mutations in 5 genes (*TP53, NOTCH1, CDKN2A, CASP8, PTEN*) from a panel of the 14 most frequently mutated genes (*CASP8, CDKN2A, FAT1, FBXW7, HRAS, IRF6, MLL2, NOTCH1, NSD1, PTEN, PIK3CA, RB1, TP53, TP63*) (unpublished results). Gene selection was based on an independent analysis of TCGA data on HNSCC using MutsigCV algorithm complemented with the list of the most frequently mutated genes reported in the literature.

To evaluate detection of ctDNA mutations without previous knowledge of tumor mutational status, we decided to sequence the entire coding region of *TP53*, as it is the most frequently mutated gene in HNSCC [25, 26, 47]. We selected 37 cases diagnosed as HNSCC from the LA study, all late stage (III and IV), from which tumor, plasma and oral rinse samples were available. A total of 49 hospital based controls from the same study with plasma and oral rinses were selected and matched by sex, age, smoking and alcohol status. Clinical and epidemiological characteristics for all Argentinian cases and controls are described in Supplementary Table 3.

### Sample preparation and DNA extraction

All cases had biological samples collected at diagnosis and before any treatment [41–43]. When feasible, two 10-ml samples of blood were collected. A portion of the blood was centrifuged for 10 min at 2000 rpm and white blood cells, red blood cells, and plasma were obtained. All samples were stored locally at −80°C (or at least −70°C depending on type of freezer). The amount of time between sample collection and freezing did not exceed 12 h.

Oral rinses were collected by performing gentle strokes in predefined areas including the right and left buccal mucosa, right, left and dorsal side of the tongue, and inside of the upper and lower lip. Cells from brushes were suspended in tubes containing phosphate-buffered saline (PBS). Participants subsequently gargled with saline, and the resulting suspension was added to the same tube. Samples were centrifuged at 3000 g for 10 minutes. Supernatant was discarded leaving ∼1,0ml to resuspend the pellet in. Samples were frozen at −70°C until shipment to Lyon (France).

Biopsy specimens were placed in liquid nitrogen or in freezers at −70°C, always within 8 hours of collection. Specimens were shipped on dry ice to Lyon, where they were stored at −70°C.

Fresh tumor tissue underwent pathological review in order to select samples with a minimum of 60% tumor cellularity for DNA extraction. Tumor DNA was extracted using the DNeasy Blood & Tissue Kit (Qiagen). cfDNA was extracted from 0,6-2,1 mL of plasma and from 1,6 to 2,0 mL of oral rinses using the QIAamp DNA Circulating Nucleic Acid kit (Qiagen) and following manufacturer's instructions. cfDNA was eluted into 100 μL of elution buffer and quantified with the Qubit DNA high-sensitivity assay kit (Invitrogen Corporation). Details regarding plasma and oral rinse volume and amount of cfDNA for all cases are included in the Supplementary Table 4.

### Primer design and amplification of targets

Twenty-one amplicons of 150 bp in size were designed (Eurofins Genomics Ebersberg, Germany) to cover targeted regions from the 5 selected genes, where mutations were identified in HNSCC fresh tumor samples. A validated in-house protocol was used to set up PCRs in 20μL reaction volume, containing 5 ng cfDNA, 60 nM of primer pool and 0,73 μL of High fidelity HotStarTaq enzyme. Amplification was carried out in DNA engine Tetrad 2 Peltier Thermal Cycler (BIORAD) as follows: 15 min at 95°C and 30 cycles of 15 seconds at 95°C and 2 min at specific annealing temperature and 10 min at 72°C. Primer sequences and amplification conditions are described in Supplementary Table 5. Primer design and amplification conditions for the *TP53* mutation discovery assays followed the procedures previously described [18].

### Library preparation and sequencing in an ion torrent^TM^ proton sequencer

Both targeted and discovery ctDNA sequencing assays followed the same library preparation and sequencing protocol previously described [18]. The following sequencing quality controls were applied: Reads with a mapping quality below 20 were excluded from subsequent analysis. An on-target coverage cut off of 400X for tumor and 3000X for plasma/oral rinses was used to select libraries. Additionally, libraries for which the on-target median coverage was significantly lower in comparison to the other libraries sequenced in the same batch were excluded. On-target median coverage for both libraries is shown in Supplementary Table 2.

### Sequencing data analyses and validation of detected mutations

For the calling of variants, we used *Needlestack*, an ultra-sensitive variant caller, which estimates the distribution of sequencing errors across multiple samples to reliably identify variants present in very low proportion (https://github.com/IARCbioinfo/needlestack). A detail description of the *Needlestack* variant caller has been previously published [18, 48]. Variant calls were annotated using ANNOVAR [49] and only *TP53* mutations reported in COSMIC-76 that were indels, nonsense, splicing, or missense variants reported as deleterious in SIFT or Polyphen, were kept for subsequent analyses. OncoPrinter and MutationMapper tools were used for visualization of mutational data [50, 51].

### Technical duplication and false positive filtering

To account for the sequencing errors and the potential false positive calls in the *TP53* discovery assay, additional quality control tests were undertaken: The whole process from PCR amplification, library preparation, and sequencing for each sample (tumor, plasma and oral rinses) was carried out in duplicate. Each technical duplicate pair was assessed independently on separate plates to avoid sample contamination.

When calling *TP53* mutations in cases and controls, *Needlestack* models de distribution of recurring sequencing errors. In some cases, some rare, random errors generated by the DNA polymerase during sequencing will be identified as variants (potential false positives). However, such errors will be generally specific to a particular library preparation and/or library sequencing. To filter out these rare errors, only mutations called in the two independent technical duplicates were considered valid and included in subsequent analyses. Additionally, we identified and excluded a few genomic positions having a particularly high proportion (>10%) of false positive calls (i.e. ‘variants’ with allelic fractions higher than the estimated sequencing error rates, but not replicable in two independent libraries).

### Statistical analyses

Survival data was available for all 36 ARCAGE cases. Overall survival was calculated from cancer diagnosis to death for any cause or end of follow up (last follow up date: 30/01/2013). Follow-up was censored at 5 years, given that most cancer related events occur before that time. The Kaplan-Meier estimator was used to estimate the distribution of the 5-year survival. Multivariate Cox proportional hazard models were used to estimate hazard ratios (HRs) and their corresponding p values for ctDNA mutation presence using age, subsite, stage, smoking and alcohol status as covariates. Log-rank test was used to compare the different survival distributions. Clinical characteristics were compared between tumor samples with and without detectable *TP53* mutations with Fisher's exact test.

## SUPPLEMENTARY FIGURE AND TABLES





## Abbreviations

cfDNA: cell free DNA
ctDNA: circulating tumor DNA
HNSCC: head and neck squamous cell carcinoma
AF: allelic frequency
