# Supplementary material for: Circulating tumor DNA detection in head and neck cancer: evaluation of two different detection approaches
Source: Oncotarget. 2017 Aug 7;8(42):72621–32. doi: 10.18632/oncotarget.20004 (PMC5641157; doi:10.18632/oncotarget.20004)
Supplement: Supplementary file 1 [file oncotarget-08-72621-s001.pdf]

Circulating tumor DNA detection in head and neck cancer: evaluation of two different detection approaches

SUPPLEMENTARY MATERIALS

A)

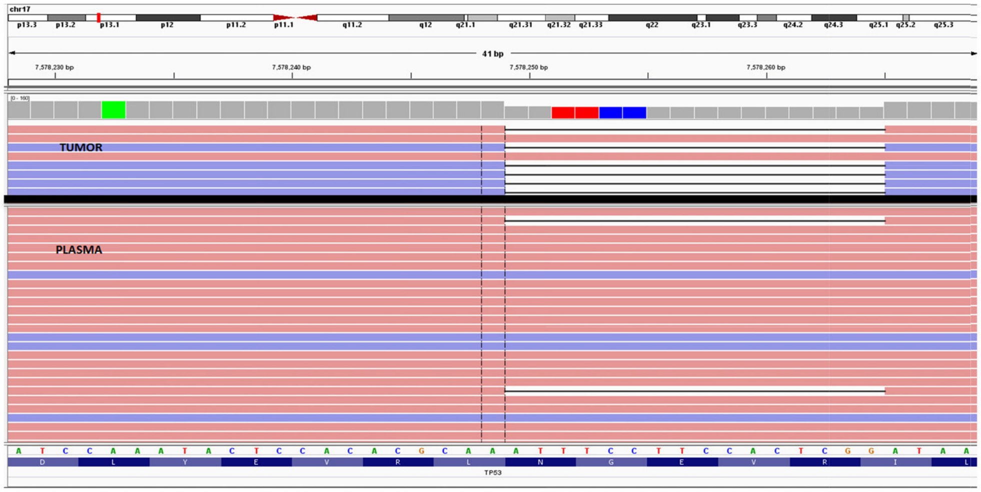

B)

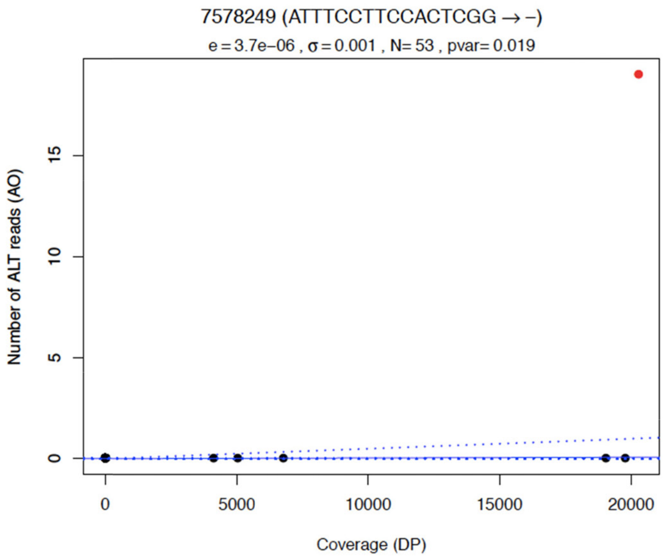

Supplementary Figure 1: A *TP53* 16-nucleotide frameshift deletion detected in a stage II oropharyngeal case. **(A)** IGV sequence alignment for both tumour and plasma. **(B)** Variant called using Needlestack's regression model of sequencing error. The blue regression line shows the estimated sequencing-error rate along with the 99%confidence interval (black dotted lines) containing samples. The outlier from the regression appears in red, and is therefore classified as positive for the given mutation.

Supplementary Table 1: Frequency of *TP53* mutations found in tumour subdivided by age, subsite, smoking and alcohol status, in 37 Argentinian HNSCC cases

Characteristic	*TP53* WT		*TP53* mutation		Total		p Value
	n	%	n	%	n	%	
**Age Group**							0,90
18 to 50	2	**22,2**	5	**17,9**	7	**18,9**	
51 to 60	3	**33,3**	11	**39,3**	14	**37,8**	
61 to 70	2	**22,2**	7	**25,0**	9	**24,3**	
>70	2	**22,2**	5	**17,9**	7	**18,9**	
**Subsite**							0,48
oral cavity	2	**22,2**	11	**39,3**	13	**35,1**	
oropharynx	1	**11,1**	4	**14,3**	5	**13,5**	
overlapping	1	**11,1**	6	**21,4**	7	**18,9**	
larynx	5	**55,6**	7	**25,0**	12	**32,4**	
**Smoking status**							0,85
Never	1	**11,1**	2	**7,14**	3	**8,1**	
Former	1	**11,1**	5	**17,9**	6	**16,2**	
Current	7	**77,8**	21	**75,0**	28	**75,7**	
**Alcohol status**							0,69
Non-drinker	1	**11,1**	5	**17,9**	6	**16,2**	
Former	0	**0,0**	4	**14,3**	4	**10,8**	
Current	8	**88,9**	19	**67,9**	27	**73,0**	
**Total**	9		28		37		

Supplementary Table 2: Description of *TP53* mutations found in tumour, plasma and oral rinses of 37 Argentinian HNSCC patients with the mutations' allelic fractions detected in the two libraries

See Supplementary File 1

Supplementary Table 3: Clinical and epidemiological description of 37 Argentinian HNSCC patients and 49 hospital based controls

Characteristic	Controls		Cases	
	**n**	**%**	**n**	**%**
	49	**100**	37	**100**
**Sex**				
Male	36	**73,5**	32	**86,5**
Female	13	**26,5**	5	**13,5**
**Age group**				
18 to 50	13	**26,5**	7	**18,9**
51 to 60	16	**32,7**	14	**37,8**
61 to 70	14	**28,6**	9	**24,3**
>70	6	**12,2**	7	**18,9**
**Smoking status**				
Never smoker	12	**24,5**	3	**8,1**
Former smoker	7	**14,3**	6	**16,2**
Current smoker	30	**61,2**	28	**75,7**
**Alcohol status**				
Non-drinker	12	**24,5**	6	**16,2**
Former drinker	8	**16,3**	4	**10,8**
Current drinker	29	**58,2**	27	**73,0**
**Subsite**				
Oral cavity	NA	**NA**	13	**35,1**
Oropharynx	NA	**NA**	5	**13,5**
Larynx	NA	**NA**	12	**32,4**
Overlapping	NA	**NA**	7	**18,9**
**Stage**				
III	NA	**NA**	3	**8,1**
IV	NA	**NA**	34	**91,9**
**Disease classification, based on 2016 ICD-10-CM Codes**				
Certain infectious and parasitic diseases	1	**2,0**	NA	**NA**
Diseases of the circulatory system	1	**2,0**	NA	**NA**
Diseases of the digestive system	7	**14,3**	NA	**NA**
Diseases of the skin and subcutaneous tissue	1	**2,0**	NA	**NA**
Diseases of the musculoskeletal system and connective tissue	10	**20,4**	NA	**NA**
Diseases of the genitourinary system	11	**22,5**	NA	**NA**
Symptoms, signs and abnormal clinical and laboratory findings, not elsewhere classified	7	**14,3**	NA	**NA**
Injury, poisoning and certain other consequences of external causes	11	**22,5**	NA	**NA**

Supplementary Table 4: Overview of the amount of cfDNA extracted per sample type (tumour, plasma, oral rinses) in both studies

Case ID	Project	Country	Total quantity cfDNA (ng) plasma	Plasma volume (ml)	Case ID	Project	Country	Total quantity DNA (ng) tumor	Total quantity cfDNA (ng) plasma	Plasma volume (ml)	Total quantity DNA (ng) oral rinses	Oral rinse volume (ml)
ARC051	ARC	Czech Republic	97,55	1,40	ARG322	LA	Argentina	3520,31	130,50	0,60	4087,79	1,60
ARC056	ARC	Czech Republic	71,36	1,20	ARG338	LA	Argentina	3074,49	121,05	1,80	5060,57	1,90
ARC062	ARC	Czech Republic	118,31	1,00	ARG355	LA	Argentina	5748,20	41,11	1,80	5650,44	2,00
ARC066	ARC	Czech Republic	73,21	1,20	ARG358	LA	Argentina	3301,36	399,00	2,00	5040,85	2,00
ARC203	ARC	Czech Republic	75,92	0,80	ARG360	LA	Argentina	11707,34	76,76	1,70	4896,89	2,00
ARC216	ARC	Czech Republic	81,98	0,80	ARG363	LA	Argentina	12093,80	62,51	1,70	1342,38	2,00
ARC238	ARC	Czech Republic	132,15	1,20	ARG374	LA	Argentina	1067,37	85,66	1,30	3672,39	2,00
ARC271	ARC	Czech Republic	82,88	1,50	ARG381	LA	Argentina	770,56	322,63	1,90	2141,93	2,00
ARC002	ARC	Greece	25,09	0,80	ARG391	LA	Argentina	1211,10	83,92	1,80	5579,76	1,80
ARC006	ARC	Greece	28,23	0,90	ARG397	LA	Argentina	539,62	65,74	1,20	4131,93	2,00
ARC186	ARC	Greece	9,82	1,00	ARG404	LA	Argentina	6446,51	77,64	1,70	4779,60	2,00
ARC009	ARC	Italy	22,14	1,50	ARG406	LA	Argentina	6375,36	86,70	1,70	4140,25	2,00
ARC016	ARC	Italy	10,82	1,00	ARG408	LA	Argentina	890,71	528,91	2,00	3596,19	1,90
ARC023	ARC	Italy	11,48	1,00	ARG411	LA	Argentina	1169,02	224,79	1,70	3039,77	2,00
ARC031	ARC	Italy	10,81	0,90	ARG416	LA	Argentina	719,33	587,01	1,80	3584,83	2,00
ARC032	ARC	Italy	31,59	1,00	ARG418	LA	Argentina	14527,42	79,55	2,00	4503,89	1,80
ARC039	ARC	Italy	12,71	0,60	ARG428	LA	Argentina	10388,78	443,56	1,60	5515,75	2,00
ARC049	ARC	Italy	17,14	1,00	ARG429	LA	Argentina	6296,08	103,41	1,80	4115,59	2,00
ARC065	ARC	Italy	6,19	0,90	ARG435	LA	Argentina	2698,19	209,87	1,80	5651,79	2,00
ARC088	ARC	Italy	21,67	0,70	ARG466	LA	Argentina	27630,49	1565,36	1,80	905,07	2,00
ARC092	ARC	Italy	9,37	1,00	ARG468	LA	Argentina	3050,29	348,51	1,70	5775,07	2,00
ARC094	ARC	Italy	10,44	1,20	ARG472	LA	Argentina	939,91	141,93	1,00	1284,78	2,00
ARC102	ARC	Italy	13,88	1,00	ARG483	LA	Argentina	788,25	75,16	1,70	5133,68	2,00
ARC118	ARC	Italy	51,05	1,20	ARG490	LA	Argentina	1860,01	592,27	1,80	4529,98	2,00
ARC182	ARC	Italy	13,53	1,20	ARG495	LA	Argentina	1928,73	42,60	1,90	5791,12	1,80
ARC206	ARC	Italy	13,79	1,00	ARG498	LA	Argentina	2092,17	264,86	1,70	5713,99	1,90
ARC214	ARC	Italy	14,00	1,20	ARG499	LA	Argentina	1100,51	79,94	1,80	5343,32	1,80
ARC246	ARC	Italy	7,72	0,90	ARG508	LA	Argentina	539,62	69,02	1,30	3530,43	2,00
ARC259	ARC	Italy	6,91	1,00	ARG533	LA	Argentina	2518,89	99,96	1,70	3172,61	1,80
ARC264	ARC	Italy	9,64	1,00	ARG540	LA	Argentina	10837,04	24,04	1,80	3945,90	2,00
ARC268	ARC	Italy	20,86	1,00	ARG541	LA	Argentina	7521,33	112,71	1,70	2039,18	1,80
ARC282	ARC	Italy	14,07	0,70	ARG559	LA	Argentina	2745,56	95,02	1,80	328,69	2,00
ARC292	ARC	Italy	13,83	1,50	ARG569	LA	Argentina	1481,68	68,18	1,60	4369,63	2,00
ARC307	ARC	Italy	12,85	1,00	ARG574	LA	Argentina	1807,36	87,46	1,80	4040,81	2,00
ARC051	ARC	Italy	26,08	1,80	ARG583	LA	Argentina	1040,74	70,43	2,00	4875,94	2,00
ARC057	ARC	Italy	37,13	1,20	ARG603	LA	Argentina	1732,55	38,39	1,90	4331,51	2,00
					ARG606	LA	Argentina	2377,39	47,76	2,00	5293,88	2,00

Supplementary Table 5: Primer sequences and PCR conditions for the targeted sequencing assay

See Supplementary File 1
