# Supplementary material for: Circulating tumor DNA detection in head and neck cancer: evaluation of two different detection approaches
Source: Oncotarget. 2017 Aug 7;8(42):72621–32. doi: 10.18632/oncotarget.20004 (PMC5641157; doi:10.18632/oncotarget.20004)
Supplement: Supplementary file 2 [file oncotarget-08-72621-s002.docx]

**Supplementary TableS2:** **Description of *TP53* mutations found in tumour, plasma and oral rinses of 37 Argentinian HNSCC patients with the mutations’ allelic fractions detected in the two libraries.**

| **Case ID** | **Sample type** | **Coverge Library1** | **Coverage Library2** | **Allelic Frequency Library1** | **Allelic Frequency Library2** | **Chromosome** | **Start** | **End** | **Ref** | **Alt** | **Mutation Function** | **Cosmic-76** | **Hgvs Protein Change** | **Transactivation Class** |
| --- | --- | --- | --- | --- | --- | --- | --- | --- | --- | --- | --- | --- | --- | --- |
| ARG322 | Oral Rinse | 20334 | 23698 | 0,005 | 0,007 | chr17 | 7579528 | 7579528 | C | T | nonsense | ID=COSM3712586 | p.Trp53* | NA |
| ARG322 | Tumor | 2115 | 1650 | 0,391 | 0,393 | chr17 | 7578394 | 7578394 | T | A | missense | ID=COSM129845 | p.His179Leu | partially functional |
| ARG338 | Oral Rinse | 17510 | 13144 | 0,003 | 0,003 | chr17 | 7577124 | 7577124 | C | T | missense | ID=COSM10891 | p.Val272Met | non-functional |
| ARG338 | Oral Rinse | 41395 | 41218 | 0,001 | 0,001 | chr17 | 7577551 | 7577551 | C | A | missense | ID=COSM1646853 | p.Gly244Cys | non-functional |
| ARG338 | Oral Rinse | 22163 | 20148 | 0,009 | 0,006 | chr17 | 7578190 | 7578190 | T | C | missense | ID=COSM99718 | p.Tyr220Cys | non-functional |
| ARG338 | Oral Rinse | 22396 | 20457 | 0,001 | 0,003 | chr17 | 7578203 | 7578203 | C | A | missense | ID=COSM1386669 | p.Val216Leu | non-functional |
| ARG338 | Oral Rinse | 24140 | 22264 | 0,005 | 0,003 | chr17 | 7578266 | 7578266 | T | A | missense | ID=COSM129840 | p.Ile195Phe | non-functional |
| ARG338 | Oral Rinse | 24301 | 22463 | 0,001 | 0,001 | chr17 | 7578271 | 7578271 | T | G | missense | ID=COSM131461 | p.His193Pro | non-functional |
| ARG338 | Tumor | 21935 | 22901 | 0,329 | 0,345 | chr17 | 7577551 | 7577551 | C | A | missense | ID=COSM1646853 | p.Gly244Cys | non-functional |
| ARG338 | Tumor | 2878 | 2639 | 0,261 | 0,272 | chr17 | 7578406 | 7578406 | C | T | missense | ID=COSM3355994 | p.Arg175His | non-functional |
| ARG355 | Tumor | 3303 | 4410 | 0,477 | 0,468 | chr17 | 7578536 | 7578536 | T | C | missense | ID=COSM10813 | p.Lys132Glu | non-functional |
| ARG358 | Oral Rinse | 7374 | 12471 | 0,030 | 0,027 | chr17 | 7577094 | 7577094 | G | A | missense | ID=COSM3378339 | p.Arg282Trp | non-functional |
| ARG358 | Oral Rinse | 7905 | 11719 | 0,009 | 0,011 | chr17 | 7578181 | 7578181 | G | A | missense | ID=COSM12193 | p.Pro223Leu | partially functional |
| ARG358 | Oral Rinse | 9656 | 15124 | 0,002 | 0,002 | chr17 | 7578272 | 7578272 | G | C | missense | ID=COSM251416 | p.His193Asp | non-functional |
| ARG358 | Tumor | 5277 | 5572 | 0,627 | 0,606 | chr17 | 7578536 | 7578536 | T | C | missense | ID=COSM10813 | p.Asn131del | NA |
| ARG360 | Tumor | 18325 | 21164 | 0,177 | 0,178 | chr17 | 7577548 | 7577548 | C | T | missense | ID=COSM1640833 | p.Gly245Ser | non-functional |
| ARG360 | Tumor | 4730 | 6179 | 0,165 | 0,160 | chr17 | 7578208 | 7578208 | T | C | missense | ID=COSM307279 | p.His214Arg | non-functional |
| ARG363 | Tumor | 6713 | 3631 | 0,461 | 0,503 | chr17 | 7578280 | 7578280 | G | A | missense | ID=COSM1386775 | p.Pro190Leu | partially functional |
| ARG381 | Tumor | 9877 | 5197 | 0,010 | 0,003 | chr17 | 7578271 | 7578271 | T | G | missense | ID=COSM131461 | p.His193Pro | non-functional |
| ARG381 | Tumor | 6041 | 2860 | 0,007 | 0,007 | chr17 | 7579350 | 7579350 | A | C | missense | ID=COSM165070 | p.Phe113Val | non-functional |
| ARG397 | Tumor | 8232 | 6682 | 0,443 | 0,436 | chr17 | 7577094 | 7577094 | G | A | missense | ID=COSM3378339 | p.Arg282Trp | non-functional |
| ARG404 | Tumor | 3523 | 5078 | 0,313 | 0,353 | chr17 | 7578517 | 7578517 | G | A | missense | ID=COSM288785 | p.Ala138Val | partially functional |
| ARG408 | Oral Rinse | 8461 | 11758 | 0,003 | 0,002 | chr17 | 7577025 | 7577025 | T | A | nonsense | ID=COSM43773 | p.Lys305* | NA |
| ARG408 | Oral Rinse | 6036 | 4815 | 0,014 | 0,015 | chr17 | 7578406 | 7578406 | C | T | missense | ID=COSM3355994 | p.Arg175His | non-functional |
| ARG408 | Tumor | 8945 | 9956 | 0,576 | 0,607 | chr17 | 7578190 | 7578190 | T | C | missense | ID=COSM99718 | p.Tyr220Cys | non-functional |
| ARG411 | Oral Rinse | 13300 | 13548 | 0,056 | 0,049 | chr17 | 7577094 | 7577094 | G | A | missense | ID=COSM3378339 | p.Arg282Trp | non-functional |
| ARG411 | Tumor | 9655 | 7400 | 0,394 | 0,375 | chr17 | 7577094 | 7577094 | G | A | missense | ID=COSM3378339 | p.Arg282Trp | non-functional |
| ARG416 | Tumor | 420 | 575 | 0,402 | 0,358 | chr17 | 7578479 | 7578479 | G | T | missense | ID=COSM43911 | p.Pro151Thr | non-functional |
| ARG418 | Tumor | 8663 | 8415 | 0,593 | 0,555 | chr17 | 7577090 | 7577090 | C | G | missense | ID=COSM10743 | p.Arg283Pro | non-functional |
| ARG428 | Oral Rinse | 30877 | 28718 | 0,034 | 0,029 | chr17 | 7577580 | 7577580 | T | C | missense | ID=COSM3388193 | p.Tyr234Cys | non-functional |
| ARG435 | Oral Rinse | 4519 | 5906 | 0,006 | 0,007 | chr17 | 7578394 | 7578394 | T | A | missense | ID=COSM129845 | p.His179Leu | partially functional |
| ARG435 | Tumor | 9678 | 10913 | 0,157 | 0,151 | chr17 | 7579528 | 7579528 | C | T | nonsense | ID=COSM3712586 | p.Trp53* | NA |
| ARG472 | Tumor | 7758 | 5938 | 0,042 | 0,045 | chr17 | 7577141 | 7577141 | C | T | missense | ID=COSM10867 | p.Gly266Glu | non-functional |
| ARG483 | Oral Rinse | 3652 | 5295 | 0,042 | 0,039 | chr17 | 7578536 | 7578536 | T | C | missense | ID=COSM10813 | p.Lys132Glu | non-functional |
| ARG483 | Tumor | 5332 | 6763 | 0,267 | 0,270 | chr17 | 7577094 | 7577094 | G | A | missense | ID=COSM3378339 | p.Arg282Trp | non-functional |
| ARG483 | Tumor | 7674 | 8440 | 0,134 | 0,132 | chr17 | 7578181 | 7578181 | G | A | missense | ID=COSM12193 | p.Pro223Leu | partially functional |
| ARG490 | Tumor | 10961 | 11287 | 0,561 | 0,560 | chr17 | 7576891 | 7576891 | T | A | nonsense | ID=COSM220789 | p.Lys319* | NA |
| ARG490 | Tumor | 9161 | 9587 | 0,016 | 0,013 | chr17 | 7578227 | 7578227 | C | A | missense | ID=COSM45734 | p.Asp208Tyr | non-functional |
| ARG490 | Tumor | 17224 | 17728 | 0,009 | 0,015 | chr17 | 7579521 | 7579521 | C | A | nonsense | ID=COSM126990 | p.Glu56* | NA |
| ARG495 | Oral Rinse | 10964 | 8462 | 0,011 | 0,014 | chr17 | 7578280 | 7578280 | G | A | missense | ID=COSM1386775 | p.Pro190Leu | partially functional |
| ARG495 | Plasma | 28478 | 42914 | 0,004 | 0,007 | chr17 | 7578410 | 7578410 | T | A | missense | ID=COSM131454 | p.Arg174Trp | non-functional |
| ARG495 | Tumor | 9402 | 10487 | 0,023 | 0,022 | chr17 | 7576891 | 7576891 | T | A | nonsense | ID=COSM220789 | p.Lys319* | NA |
| ARG498 | Tumor | 10509 | 7723 | 0,062 | 0,065 | chr17 | 7578190 | 7578190 | T | C | missense | ID=COSM99718 | p.Tyr220Cys | non-functional |
| ARG498 | Tumor | 10667 | 7857 | 0,006 | 0,010 | chr17 | 7578236 | 7578236 | A | C | missense | ID=COSM1564190 | p.Tyr205Asp | non-functional |
| ARG498 | Tumor | 960 | 711 | 0,027 | 0,024 | chr17 | 7578457 | 7578457 | C | A | missense | ID=COSM10714 | p.Arg158Leu | non-functional |
| ARG499 | Oral Rinse | 30720 | 32243 | 0,218 | 0,211 | chr17 | 7579900 | 7579900 | G | A | nonsense | ID=COSM44191 | p.Gln5* | NA |
| ARG499 | Tumor | 20300 | 17994 | 0,003 | 0,002 | chr17 | 7577518 | 7577518 | T | - | frameshift deletion | ID=COSM46339 | p.Ile255fs | NA |
| ARG508 | Tumor | 20907 | 18981 | 0,249 | 0,238 | chr17 | 7577580 | 7577580 | T | C | missense | ID=COSM3388193 | p.Tyr234Cys | non-functional |
| ARG540 | Plasma | 32749 | 26214 | 0,004 | 0,006 | chr17 | 7578410 | 7578410 | T | A | missense | ID=COSM131454 | p.Arg174Trp | non-functional |
| ARG541 | Tumor | 4777 | 4380 | 0,099 | 0,100 | chr17 | 7577120 | 7577120 | C | T | missense | ID=COSM99729 | p.Arg273His | non-functional |
| ARG559 | Oral Rinse | 19253 | 38561 | 0,002 | 0,001 | chr17 | 7578253 | 7578253 | C | A | missense | ID=COSM44140 | p.Gly199Val | non-functional |
| ARG559 | Oral Rinse | 12004 | 32374 | 0,001 | 0,002 | chr17 | 7579366 | 7579366 | G | T | nonsense | ID=COSM213589 | p.Tyr107* | NA |
| ARG559 | Tumor | 2400 | 2386 | 0,569 | 0,561 | chr17 | 7578412 | 7578412 | A | C | missense | ID=COSM44383 | p.Val173Gly | non-functional |
| ARG569 | Oral Rinse | 31707 | 22133 | 0,003 | 0,003 | chr17 | 7577550 | 7577550 | C | T | missense | ID=COSM1646854 | p.Gly244Asp | non-functional |
| ARG569 | Oral Rinse | 33058 | 14400 | 0,003 | 0,003 | chr17 | 7578235 | 7578235 | T | C | missense | ID=COSM99631 | p.Tyr205Cys | non-functional |
| ARG569 | Tumor | 7174 | 4472 | 0,032 | 0,048 | chr17 | 7577018 | 7577018 | C | T | splicing | ID=COSM213111 | NA | NA |
| ARG574 | Oral Rinse | 12303 | 12195 | 0,017 | 0,017 | chr17 | 7577081 | 7577081 | T | C | missense | ID=COSM3958795 | p.Glu286Gly | non-functional |
| ARG574 | Tumor | 6099 | 4784 | 0,217 | 0,203 | chr17 | 7577081 | 7577081 | T | C | missense | ID=COSM3958795 | p.Glu286Gly | non-functional |
| ARG576 | Oral Rinse | 5105 | 4076 | 0,004 | 0,005 | chr17 | 7579350 | 7579350 | A | T | missense | ID=COSM3773320 | p.Phe113Ile | non-functional |
| ARG583 | Oral Rinse | 6321 | 7053 | 0,025 | 0,026 | chr17 | 7578413 | 7578413 | C | A | missense | ID=COSM3723936 | p.Val173Leu | non-functional |
| ARG583 | Oral Rinse | 7096 | 8027 | 0,204 | 0,206 | chr17 | 7579900 | 7579900 | G | A | nonsense | ID=COSM44191 | p.Gln5* | NA |
| ARG583 | Plasma | 36766 | 33699 | 0,002 | 0,005 | chr17 | 7578413 | 7578413 | C | A | missense | ID=COSM3723936 | p.Val173Leu | non-functional |
| ARG583 | Tumor | 2554 | 2974 | 0,255 | 0,235 | chr17 | 7578413 | 7578413 | C | A | missense | ID=COSM3723936 | p.Val173Leu | non-functional |
| ARG603 | Tumor | 7294 | 5613 | 0,001 | 0,002 | chr17 | 7578535 | 7578537 | TTG | - | nonframeshift deletion | ID=COSM1290769 | p.Lys132Glu | non-functional |
| ARG606 | Tumor | 22544 | 18974 | 0,728 | 0,749 | chr17 | 7577539 | 7577539 | G | A | missense | ID=COSM3388183 | p.Arg248Trp | non-functional |

**Supplementary TableS5: Primer sequences and PCR conditions for the targeted sequencing assay.**

| **GENE-PRIMER REGION** | **PRIMER SEQUENCE** | **PRODUCT SIZE** | **EXON** | **ANNEALING T(°C)** |
| --- | --- | --- | --- | --- |
| **TP53-ex4-1-F** | TCTGACTGCTCTTTTCACCCA | 127 | 4 | 51,2 |
| **TP53-ex4-1-R** | TCTGGGAGCTTCATCTGGAC |  |  |  |
| **TP53-ex4-4-F** | TCCTGGCCCCTGTCATCTT | 160 | 4 | 57,2 |
| **TP53-ex4-4-R** | ATACGGCCAGGCATTGAAGT |  |  |  |
| **TP53-ex5-1-F** | TTCAACTCTGTCTCCTTCCTCTTC | 135 | 5 | 59,7 |
| **TP53-ex5-1-R** | ATGGCGCGGACGCGGGT |  |  |  |
| **TP53-ex5-2-F** | AGCTGTGGGTTGATTCCA | 162 | 5 | 64,8 |
| **TP53-ex5-2-R** | AGCCCTGTCGTCTCTCCA |  |  |  |
| **TP53-ex6-1-F** | GCCTCTGATTCCTCACTGAT | 108 | 6 | 48,1 |
| **TP53-ex6-1-R** | CGAAAAGTGTTTCTGTCATCC |  |  |  |
| **TP53-ex6-2-F** | TTGCGTGTGGAGTATTTGGA | 102 | 6 | 52,9 |
| **TP53-ex6-2-R** | CCTCCCAGAGACCCCAGTT |  |  |  |
| **TP53-ex7-1-F** | CTGCTTGCCACAGGTCTCCCC | 127 | 7 | 55,1 |
| **TP53-ex7-1-R** | ATGCCGCCCATGCAGGAA |  |  |  |
| **TP53-ex7-2-F** | GTTGGCTCTGACTGTACCACCATC | 137 | 7 | 55,5 |
| **TP53-ex7-2-R** | TGTGCAGGGTGGCAAGTG |  |  |  |
| **TP53-ex8-1-F** | TCCTTACTGCCTCTTGCTTCTCTT | 115 | 8 | 53 |
| **TP53-ex8-1-R** | TCCTCTGTGCGCCGGTCT |  |  |  |
| **TP53-ex8-2-F** | CGTGTTTGTGCCTGTCCTG | 156 | 8 | 59,6 |
| **TP53-ex8-2-R** | AGGCATAACTGCACCCTTGG |  |  |  |
| **TP53-ex10-F** | ACTTCTCCCCCTCCTCTGTT | 156 | 10 | 62,2 |
| **TP53-ex10-R** | GGAAGGGGCTGAGGTCACT |  |  |  |
| **NOTCH1-ex33-F** | CAGCTGGTCTCCAACCTACC | 120 | 33 | 58 |
| **NOTCH1-ex33-R** | TTGTTAGCCCCGTTCTTCAG |  |  |  |
| **NOTCH1-ex12-F** | AACTGCGAGATCAACCTGGA | 149 | 12 | 58 |
| **NOTCH1-ex12-R** | TCTGAGCACAGTGCAGTCA |  |  |  |
| **NOTCH1-ex9-F** | TTCGTTTCTGTCCCAAGTCC | 134 | 9 | 58 |
| **NOTCH1-ex9-R** | GCTGCTGGCACACTCGTCT |  |  |  |
| **NOTCH1-ex21-F** | TACTGTGAGGACCTGGTGGAC | 123 | 21 | 57,5 |
| **NOTCH1-ex21-R** | GGCCACAACCCTTACCCTA |  |  |  |
| **NOTCH1-ex31-F** | CCGCTACTCACGCTCTGAT | 129 | 31 | 59 |
| **NOTCH1-ex31-R** | ACCTGGAAGACACCTTGTGC |  |  |  |
| **NOTCH1-ex34-F** | GCCTGTGGAAGCAAGGAG | 145 | 34 | 56 |
| **NOTCH1-ex34-R** | AGGCCACGTCTGACAGGTAG |  |  |  |
| **NOTCH1-ex32-F** | GAGGCATCGGTGTACGTCTG | 123 | 32 | 58 |
| **NOTCH1-ex32-R** | AGGATCAGTGGCGTCGTG |  |  |  |
| **NOTCH1-ex07-F** | ATCAGCAACCCCTGTAACGA | 130 | 7 | 58 |
| **NOTCH1-ex07-R** | TACCCAGCGAGCACTCATC |  |  |  |
| **NOTCH1-ex08-F** | GGCAAGTGCATCAACACG | 135 | 8 | 58 |
| **NOTCH1-ex08-R** | AATCTGGTCCAGGCAGGT |  |  |  |
| **NOTCH1-ex08-F** | CACCTGCCTGGACCAGATT | 136 | 8 | 58 |
| **NOTCH1-ex08-R** | CGGAAGCAACCCACAGAT |  |  |  |
| **CDKN2A-ex02-F** | CTGTTCTCTCTGGCAGGTCA | 157 | 2 | 59 |
| **CDKN2A-ex02-R** | CAGCACCACCAGCGTGTC |  |  |  |
| **CDKN2A-ex02-F** | GACACGCTGGTGGTGCTG | 157 | 2 | 57 |
| **CDKN2A-ex02-R** | GGGCATGGTTACTGCCTCT |  |  |  |
| **CDKN2A-ex01-F** | ATGGAGCCTTCGGCTGACT | 141 | 1 | 56 |
| **CDKN2A-ex01-R** | AGACCCTCTACCCACCTGGA |  |  |  |
| **CASP8-ex03-F** | GATGAACAAGCCAGCAAATG | 150 | 3 | 58 |
| **CASP8-ex03-R** | GGCCAGATCTTCACTGTCCA |  |  |  |
| **CASP8-ex03-F** | AGGAGCTGCTCTTCCGAAT | 130 | 3 | 58 |
| **CASP8-ex03-R** | GTTTCCACCCACCTGTAGGC |  |  |  |
| **CASP8-ex08-F** | GGATTTATCATCACCTCAAACG | 134 | 8 | 58 |
| **CASP8-ex08-R** | TCTGGCAAAGTGACTGGATG |  |  |  |
| **CASP8-ex06-F** | CCATGTCTCATTCTAGATGTGTCTC | 121 | 6 | 58 |
| **CASP8-ex06-R** | TGGAGGTTTCCGTCTACCTG |  |  |  |
| **CASP8-ex09-F** | GAGGAGACAGTTCATCAGTTGC | 152 | 9 | 58 |
| **CASP8-ex09-R** | AGGCATCTGTTTCCCCATGT |  |  |  |
| **PTEN-ex05-F** | GAGGTTATCTTTTTACCACAGTTGC | 150 | 5 | 58 |
| **PTEN-ex05-R** | TTCCAGCTTTACAGTGAATTGC |  |  |  |
